# Extremophilic Bacterium *Halomonas desertis* G11 as a Cell Factory for Poly-3-Hydroxybutyrate-co-3-Hydroxyvalerate Copolymer’s Production

**DOI:** 10.3389/fbioe.2022.878843

**Published:** 2022-05-23

**Authors:** Khouloud Hammami, Yasmine Souissi, Amal Souii, Awatef Ouertani, Darine El-Hidri, Marwa Jabberi, Habib Chouchane, Amor Mosbah, Ahmed Slaheddine Masmoudi, Ameur Cherif, Mohamed Neifar

**Affiliations:** ^1^ BVBGR-LR11ES31, Higher Institute of Biotechnology of Sidi Thabet (ISBST), University of Manouba, Ariana, Tunisia; ^2^ Department of Engineering, German University of Technology in Oman, Muscat, Oman; ^3^ APVA-LR16ES20, National School of Engineers of Sfax (ENIS), University of Sfax, Sfax, Tunisia

**Keywords:** *Halomonas desertis* G11, halophilic bacterium, polyhydroxyalkanoates, genome annotation, PHA synthase

## Abstract

Microbial polyhydroxyalkanoates (PHA) are biodegradable and biocompatible bio-based polyesters, which are used in various applications including packaging, medical and coating materials. In this study, an extremophilic hydrocarbonoclastic bacterium, previously isolated from saline sediment in the Tunisian desert, has been investigated for PHA production. The accumulation of intracellular PHA granules in *Halomonas desertis* G11 was detected by Nile blue A staining of the colonies. To achieve maximum PHA yield by the strain G11, the culture conditions were optimized through response surface methodology (RSM) employing a Box-Behnken Design (BBD) with three independent variables, namely, substrate concentration (1–5%), inoculum size (1–5%) and incubation time (5–15 days). Under optimized conditions, G11 strain produced 1.5 g/L (68% of DCW) of PHA using glycerol as a substrate. Application of NMR (1H and 13C) and FTIR spectroscopies showed that *H. desertis* accumulated PHA is a poly-3-hydroxybutyrate-co-3-hydroxyvalerate (PHBV). The genome analysis revealed the presence of typical structural genes involved in PHBV metabolism including *phaA*, *phaB*, *phaC*, *phaP*, *phaZ*, and *phaR*, coding for acetyl-CoA acetyltransferase, acetoacetyl-CoA reductase, class I polyhydroxyalkanoates synthases, phasin, polyhydroxyalkanoates depolymerase and polyhydroxyalkanoates synthesis repressor, respectively. Glycerol can be metabolized to 1) acetyl-CoA through the glycolysis pathway and subsequently converted to the 3HB monomer, and 2) to propionyl-CoA via the threonine biosynthetic pathway and subsequently converted to the 3HV monomer. *In silico* analysis of PhaC1 from *H. desertis* G11 indicated that this enzyme belongs to Class I PHA synthase family with a “lipase box”-like sequence (SYCVG). All these characteristics make the extremophilic bacterium *H. desertis* G11 a promising cell factory for the conversion of bio-renewable glycerol to high-value PHBV.

## Introduction

Fossil fuel-based plastics play a crucial role in modern lifestyle (Chae and An, 2018). Approximately 400 million tons of plastic were produced globally in 2018. More than 10 million metric tons of plastic ended up into the oceans during 2018 alone. An estimated 13 billion tons of plastic waste will reach the environment by 2050 in the absence of any improvements in current plastic waste management practices (Shams et al., 2021). Synthetic plastics have significant negative impacts on ecosystems, biota, environment, economy, and human health (Sharma and Chatterjee, 2017). As a result, there is a growing demand for bioplastics such as polyhydroxyalkanoates (PHA), polylactic acid, biopolyamide, biopolyethylene and biopolypropylene that provide new solutions in terms of life cycle and raw materials (Akinwumi et al., 2019). The conversion of biowastes to PHAs is considered as a possible eco-friendly alternative to petro-plastics and are currently gaining a lot of interest in the field of industrial applications and waste management (Kourmentza et al., 2017; Patel et al., 2021). Global bioplastic production has increased from 2.1 metric tons in 2020 to about 2.4 metric tons in 2022 (Dubey and Mishra, 2021) and the market opportunity for PHA bioplastic could reach US$100 million by 2024 (Surendran et al., 2020).

PHA are linear polyesters that consist of hydroxy fatty acids and are synthesized by a wide range of different Gram-positive and Gram-negative bacteria, as well as in some haloarchaeal species (Bugnicourt et al., 2014; Han et al., 2015; Kalia et al., 2021). Among the PHA producing microorganisms, the most-studied were *Pseudomonas oleovorans*, *P. aeruginosa* and *P. putida*, *Bacillus megaterium*, *B. cereus*, *Ralstonia eutropha,* and *Haloferax mediterranei* (Poli et al., 2011; Kalia et al., 2021). PHAs are usually produced when the microorganismes are cultured in the presence of excess carbon sources and limited nitrogen, phosphorus, sulfur, or oxygen (Surendran et al., 2020). They are stored as reserve foods as either inclusion bodies or within calcium (Ca) or polyphosphate complexes. In a few cases, PHA can accumulate to 90% of the total cell dry weight (CDW), which helps the microorganism survive under environmental stress conditions (Kalia et al., 2021). PHA can be classified in three main categories: short-chain-length PHA (scl-PHA) consists of 3-5 carbons atoms [3-hydroxybutyrate (3HB), and 3-hydroxyvalerate (3HV)]; medium-chain-length PHA (mcl-PHA) that consists of 6–14 carbons atoms [3-hydroxyhexanoate (3HHx), 3-hydroxyheptanoate (3HHp), and 3-hydroxytetradecanoate (3HTD)…] and also mixtures of both scl-PHA and mcl-PHA (Leong et al., 2014; Tan et al., 2014). More than 150 different monomers have been identified in PHA chains under various fermentation conditions, resulting in PHA with different characteristics (Lee et al., 2019). Due to their properties similar to those of petrochemical thermoplastics and combined properties of biodegradability and biocompatibility, PHA have gained considerable importance as promising candidates for biologically-derived plastics as well as biomaterials (Han et al., 2010, 2015, 2017; Kourmentza et al., 2017; Mitra et al., 2020).

Poly (3-hydroxybutyrate-co-3-hydroxyvalerate), PHBV, is a biopolyester with good mechanical properties and biodegradability, with broad applications in a wide variety of sectors (food and medical packaging, drug release and transport systems, hygiene products, etc.) due to its excellent properties such as absorption capacity, low cytotoxicity, piezoelectricity, and thermoplasticity compared to brittle PHB (Tan et al., 2014; Rivera-Briso and Serrano, 2018; Chen et al., 2019). As reported by Ibrahim et al. (2020), PHBV is a very promising copolymer that has great potential to replace conventional non-degradable polymers and has a great sustainability potential in circular economy development strategy. For most industrial uses of PHBV, the 3HV fraction should be at least in the range of 10–20% and usually chemical precursors such as propionate and valerate were added to obtain such 3HV fraction (Hermann-Krauss et al., 2013). Increasing 3HV content enhances the biodegradability and reduces the crystallinity and melting point of the bio-copolymer (Policastro et al., 2021). However, even though it owns environmental advantages and more suitable properties compared to other bioplastics, the main problem facing commercial production is the high production costs and low productivity rate. Therefore, the current challenge for researchers is the implementation of efficient and low cost PHBV production processes (Albuquerque and Malafaia, 2018; Patel et al., 2021 a and b; Policastro et al., 2021). Therefore, many approaches have been developed to minimize the production cost such as 1) the use of agricultural wastes and industrial by-products (e.g., fruit peels, bagasse, waste plant oils, crude glycerol, cheese whey, etc.) as inexpensive carbon sources with widespread availability and renewability (Kulkarni et al., 2015; Patel et al., 2015; de Paula et al., 2017; Ray et al., 2018:; Surendran et al., 2020), 2) the application of experimental designs and response surface methodology as statistical tools for the optimization of microbial biomass and PHA production using different cultivation modes (batch, fed-batch, and continuous fermentations) (El-malek et al., 2020; Singh et al., 2021), 3) the use of extremophilic bacteria as efficient PHA-producing strains (Cai et al., 2011; Yin et al., 2015; Kourmentza et al., 2017; Chen et al., 2018; Patel S. K. S. et al., 2021; Dubey and Mishra 2021). As an example, PHA production can be more environmentally friendly low-cost feed like using greenhouse gases (biogas) by thermophilic methanotrophs such as under non-sterile conditions (Patel S. K. S. et al., 2021).

Microbial halophiles are considered to be a promising cell factory for high-value biomolecules synthesis (extremolytes, extremozymes, biosurfactants, bioplastics, etc.) due to their unique characteristics of thriving under extremes of temperature, salinity, pH, and solvent conditions (Mitra et al., 2020). Extremely halotolerant species (up to 20% salinity) of the genus *Halomonas*, mostly associated with saline or hypersaline environments such as ocean waters and hypersaline lakes have shown PHA production performance under open and non-sterile conditions allowing the development of continuous bioprocesses without contamination (Kourmentza et al., 2017; Neifar et al., 2019; Athmika et al., 2021). The Chinese company Bluepha produces both PHB and PHBV from a halophilic *Halomonas* strain (Mitra et al., 2020). The genomes of PHA-producing extremophilic strains were studied *via* bioinformatics and genetic engineering tools for the expression of the PHA synthesis genes in non-producing strains exposing less restrictive growth and production conditions (Cai et al., 2011; Thomas et al., 2020). Policastro et al. (2021) reviewed the most relevant studies on PHBV production by mesophilic and extremophilic strains and the strategies used for costs reduction. Halophilic and halotolerant *Halomonas* strains such as *Halomonas* sp. TD-0, *Halomonas* sp. SF2003, *Halomonas* i4786, *H. pacifica* ASL10 and *H. salifodiane* ASL11 have been reported as promising candidates able to produce short chain length PHA up to 80 wt% of cell dry weight (CDW), using conventional carbon sources as well as carbonaceous by-products from food wastes (Elain et al., 2016; El-Malek et al., 2020; Thomas et al., 2020; Dubey and Mishra, 2021).

Thus, the present study investigates the ability of the halophilic bacterium *Halomonas desertis* G11, isolated from the largest salt-lake in Tunisian Sahara (Neifar et al., 2019), to directly produce PHBV from a low-cost and non-toxic substrate and without addition of PHV precursors. The study focused on the use of glycerol as a carbon substrate taking into account that it is one of the promising substrates for large-scale production of PHA due to the growing production of biodiesel as an alternative renewable energy source (Kumar et al., 2015; Ray et al., 2018; Kondaveeti et al., 2019). The culture conditions were optimized using experimental design and response surface methodologies. This work will also contribute to a better understanding of PHA metabolism and PHA synthase functionality of G11 strain by genomic and structural approaches.

## Materials and Methods

### Sources of Bacterial Strain and Genome Sequences

The extremophilic hydrocarbonoclastic bacterium *Halomonas desertis* G11 used in this study was isolated from hypersaline sediment of Chott El-Djerid of southern Tunisia (Neifar et al., 2019; Riahi et al., 2020). It has an optimum growth at 5–10% NaCl and pH 8-10. The complete genomic sequences of G11 have been deposited in GenBank under the accession number LYXG00000000. The genomic and proteomic sequence data of the strain G11 was downloaded from the Universal Protein Resource UniProt database (https://www.uniprot.org/uniprot/?query=halomonas+g11&sort=score) and the Integrated Microbial Genomes and Microbiomes (IMG/M) platform (https://img.jgi.doe.gov/cgi-bin/m/main.cgi?section=TaxonDetail&page=taxonDetail&taxon_oid=2751185866).

### Detection of PHA Accumulation in *Halomonas desertis* G11 After Nile Blue a Staining

Detection of intracellular accumulation of PHA was performed according to the Nile blue A staining method (Ostle and Holt, 1982). *Halomonas desertis* G11 was grown in modified high medium (HM) containing 10 g/L glycerol, 2 g/L yeast extract, 50 g/L NaCl; 0.25 g/L MgSO_4_·7H_2_O, 0.09 g/L CaCl_2_·2H_2_O, 0.5 g/L KCl, 0.25 g/L KH_2_PO_4_, 2 g/L granulated agar, 0.06 g/L NaBr, and Nile blue A (dissolved in dimethylsulfoxide) with a final concentration of 0.5 mg dye per L of medium (Hertadi et al., 2017). After 4 days of bacterial incubation at 30°C, the agar plates were exposed to ultraviolet light to visualize the fluorescence (Bhuwal et al., 2013; Mascarenhas and Aruna, 2017).

### Experimental Conditions for PHA Production and Recovery

The PHA production by *H. desertis* G11 was carried out in modified liquid HM medium (Hertadi et al., 2017) using glycerol as a carbon substrate (concentrations 1–5%). 250 ml shake flask cultures (100 ml working volumes) were incubated at 30°C with an orbital agitation of 120 rpm. Bacterial growth was followed by monitoring optical densities (ODs) at 600 nm wavelength. Cells were washed with distilled water and then dried until constant cell weight was achieved. After incubation, G11 cells were recovered from cultures through centrifugation at 6,000 rpm for 15 min and the biomass was used for PHA extraction. Approximately 5 ml chloroform was added to 20 mg biomass and then incubated for 24 h at 60°C. The residual bacterial biomass was separated by filtration; as described by Kucera et al. (2018). PHA was precipitated by the addition of 2× volume of methanol. The white precipitate formed was filtered, dried in an oven at 60°C under vacuum for 24 h, weighed and the yield of PHA was expressed as % PHA formed on dry cell weight basis (Kulkarni et al., 2010; Hertadi et al., 2017). The purified PHA was used for subsequent analysis.

### Optimization of PHA Production by Response Surface Methodology

**FIGURE 1:**
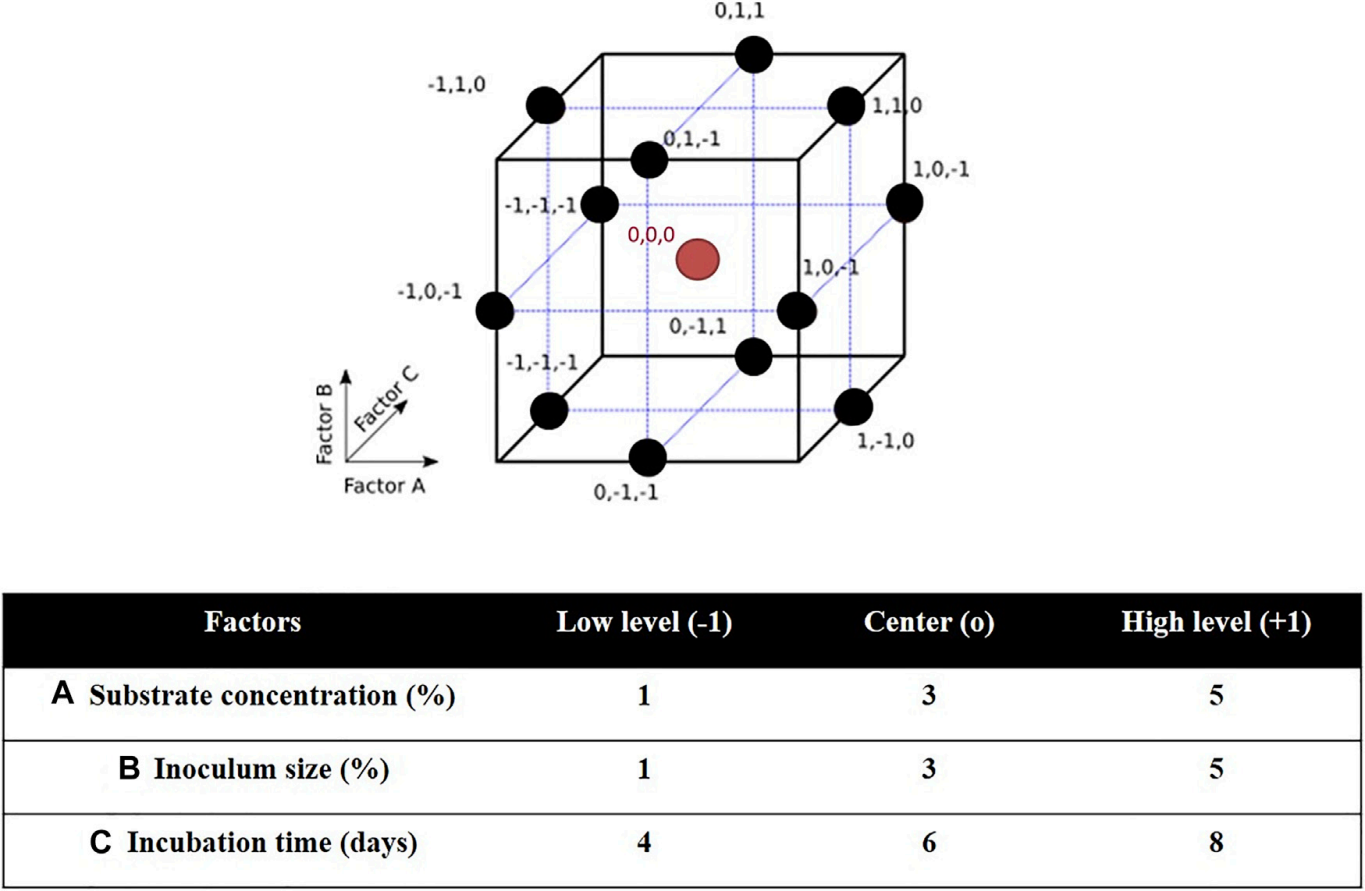
Characteristics of Box–Behnken design (BBD): BBD space for three factors and BBD experimental domain.

Three-factor three-level Box-Behnken design (BBD) and response surface methodology (RSM) were used to determine the optimal culture conditions for PHA production by *H. desertis* G11. The BBD space and experimental domain were shown in Figure 1. The BBD is a type of nearly rotatable second-order design, which means that the model has a reasonably stable distribution of the prediction variance throughout the experimental domain. As a result, it is more efficient and cost-effective than the full 3k factorial design. It requires three levels for each factor instead of five as in the central composite design (CCD), which results in fewer experimental trials, and is more convenient and less expensive than CCD. Another advantage of the BBD is that it does not contain combinations in which all factors are simultaneously set at their extreme levels. These designs are therefore useful for avoiding experiments performed under such conditions for which unsatisfactory results could be observed (Montgomery, 2005).

The relationship between the response R and the three quantitative factors A, B, and C was approximated by the following second-order polynomial equation:

$$R=e_{0}+e_{1}A+e_{2}B+e_{3}C+e_{11}A^{2}+e_{22}B^{2}+e_{33}C_{3}^{2}+e_{12}\;A_{B}+e_{13}AC+e_{23}BC$$

Where R is the predictive measured response as PHA production yield (g/L); A, B, and C represent coded values of substrate concentration (%); inoculum size (%); and incubation time (days).; e_0_ is the intercept, e_1_, e_2_, e_3_, e_11_, e_22_ and e_33_ are the regression coefficients.

The accuracy and fitness of the above model were analyzed by ANOVA and evaluated by the coefficient of determination (R^2^) and F value. The optimum levels of studied factors for maximum PHA production were obtained by solving the regression equation, analyzing the contour plots and 3D surface plots. For validation, additional experiments were performed under these optimal conditions.

### Characterization of Produced PHA by Spectroscopy Analyses

Produced PHA was analyzed by Fourier Transform Infrared (FT-IR) and Nuclear Magnetic Resonance (NMR) spectroscopies. The purified polymer (2 mg) of PHA was subjected to FTIR analysis to characterize the presence of specific functional groups. The spectrum was recorded using a FTIR spectrometer (Nicolet IR200 FT-IR) with a spectral range 4,000 and 400 cm^−1^. For ^1^H and ^13^C NMR analysis, PHA samples were dissolved in deuterated chloroform (CDCl3) and analyzed on a 400 MHz NMR spectrophotometer (Divyashree and Shamala, 2009; El-malek et al., 2020). The composition of PHB and PHV in the copolymer was calculated from the area ratio of absorption peaks in methyl groups corresponding to the HB and HV groups (1.2 and 0.9 ppm, respectively) as reported by Kulkarni et al. (2010).

### Identification of PHA Related Genes by Genomic Analysis

Genome annotation was performed using the Rapid Annotations using Subsystems Technology (RAST, http://rast.nmpdr.org/) server (Aziz et al., 2008) to identify genes involved in the metabolic pathways of PHA. The gene predictions were manually verified by BLAST searches against the protein databases NCBI (http://www.ncbi.nlm.nih.gov/) and the UniProt database (http://www.uniprot.org/).

### Phylogenetic Analysis and Classification of *H. desertis* PHA Synthase

Multiple alignments and phylogeny analysis were performed with a group of characterized PHA synthases from several PHA-producing strains selected from the UniProt database. A Neighbour Joining starting tree was generated from this alignment through Mega-X. The identification of PhaC1 box lipase consensus sequence of *H. desertis* G11 was conducted using BioEdit software using the ClustalW multiple alignment tool (Thomas et al., 2020).

### Analysis of the Primary Amino Acid Sequences and Structure Prediction of PHA Synthase PhaC1

The primary structure of the PhaC1 was analyzed using the ExPASy-ProtParam tool (Gasteiger et al., 2005). Its secondary structure was predicted using the self-optimized prediction method (SOPMA), Phyre2 (http://www.sbg.bio.ic.ac.uk/phyre2) (Kelley et al., 2015) and PredictProtein (http://www.predictprotein.org/). The 3D models of the enzymes were constructed using I-TASSER homology modeling servers (Yang and Zhang, 2015). I-TASSER generates five three-dimensional structures for each model. The coordinates of the refined model were evaluated by MolProbity (Davis et al., 2007). Models were evaluated by Coach (Yang et al., 2013), a protein-ligand binding site prediction software and visualized using the PyMOL molecular graphics system.

### Statistical Analysis

The regression analysis, estimation of the coefficient, generation and data treatments, plotting 2D and 3-D plots of BBD were performed using the experimental design software NemrodW (Mathieu et al., 2000).

## Results

### Preliminary Assessment of PHA Accumulation

The simple and highly sensitive Nile blue A staining method was used to detect PHA accumulation in growing *H. desertis* colonies. As revealed in Supplementary Figure S1, the strain G11 showed bright orange fluorescence indicating PHA accumulation.

### Experimental Design and Statistical Modeling of PHA Production

*H. desertis* G11 was able to utilize glycerol as a carbon source for growth and PHA synthesis (Table 1). BBD and RSM were applied to determine the optimal conditions for PHA production. The variables and their levels were selected based on preliminary tests and bibliographic results. The BBD along with corresponding experimental and predicted responses of PHA and biomass productions, was shown in Table 1. The following quadratic equation was obtained by regression analysis to predict PHA production by the strain G11.

$$R=0.633+0.062A+0.087B-0.525C+0.108A^{2}-0.142B^{2}+0.133C^{2}+0.050AB-0.075AC-0.025BC$$

Where, R is the PHA production yields (g/L), and A, B, and C are the factors coded values of substrate concentration (%), inoculum size (%) and incubation period (days), respectively.

**TABLE 1:** Experimental conditions of BDD in natural variables and the corresponding experimental and theoretical PHA production responses.

Run N	Coded variables			Substrate concentration (%)	Inoculum size (%)	Incubation time (days)	(PHA) (g/L)		(Biomass) (g/L)	
	A	B	C				Measured response	Estimated response	Measured response	Estimated response
1	−1.0	−1.0	0.0	1.0	1.0	8.0	0.50	0.50	3.0	3.4
2	1.0	−1.0	0.0	5.0	1.0	8.0	0.50	0.53	2.0	2.1
3	−1.0	1.0	0.0	1.0	5.0	8.0	0.60	0.57	3.5	3.4
4	1.0	1.0	0.0	5.0	5.0	8.0	0.80	0.80	5.0	4.6
5	−1.0	0.0	−1.0	1.0	3.0	4.0	1.30	1.26	2.5	2.5
6	1.0	0.0	−1.0	5.0	3.0	4.0	1.60	1.54	2.0	2.2
7	−1.0	0.0	1.0	1.0	3.0	8.0	0.30	0.36	1.5	1.2
8	1.0	0.0	1.0	5.0	3.0	8.0	0.30	0.34	1.5	1.5
9	0.0	−1.0	−1.0	3.0	1.0	5.0	1.00	1.04	3.0	2.6
10	0.0	1.0	−1.0	3.0	5.0	5.0	1.20	1.26	2.5	2.6
11	0.0	−1.0	1.0	3.0	1.0	8.0	0.10	0.04	0.5	0.4
12	0.0	1.0	1.0	3.0	5.0	8.0	0.20	0.16	2.5	2.9
13	0.0	0.0	0.0	3.0	3.0	6.0	0.60	0.63	5.0	5.0
14	0.0	0.0	0.0	3.0	3.0	6.0	0.60	0.63	5.3	5.0
15	0.0	0.0	0.0	3.0	3.0	6.0	0.70	0.63	4.7	5.0

**FIGURE 2:**
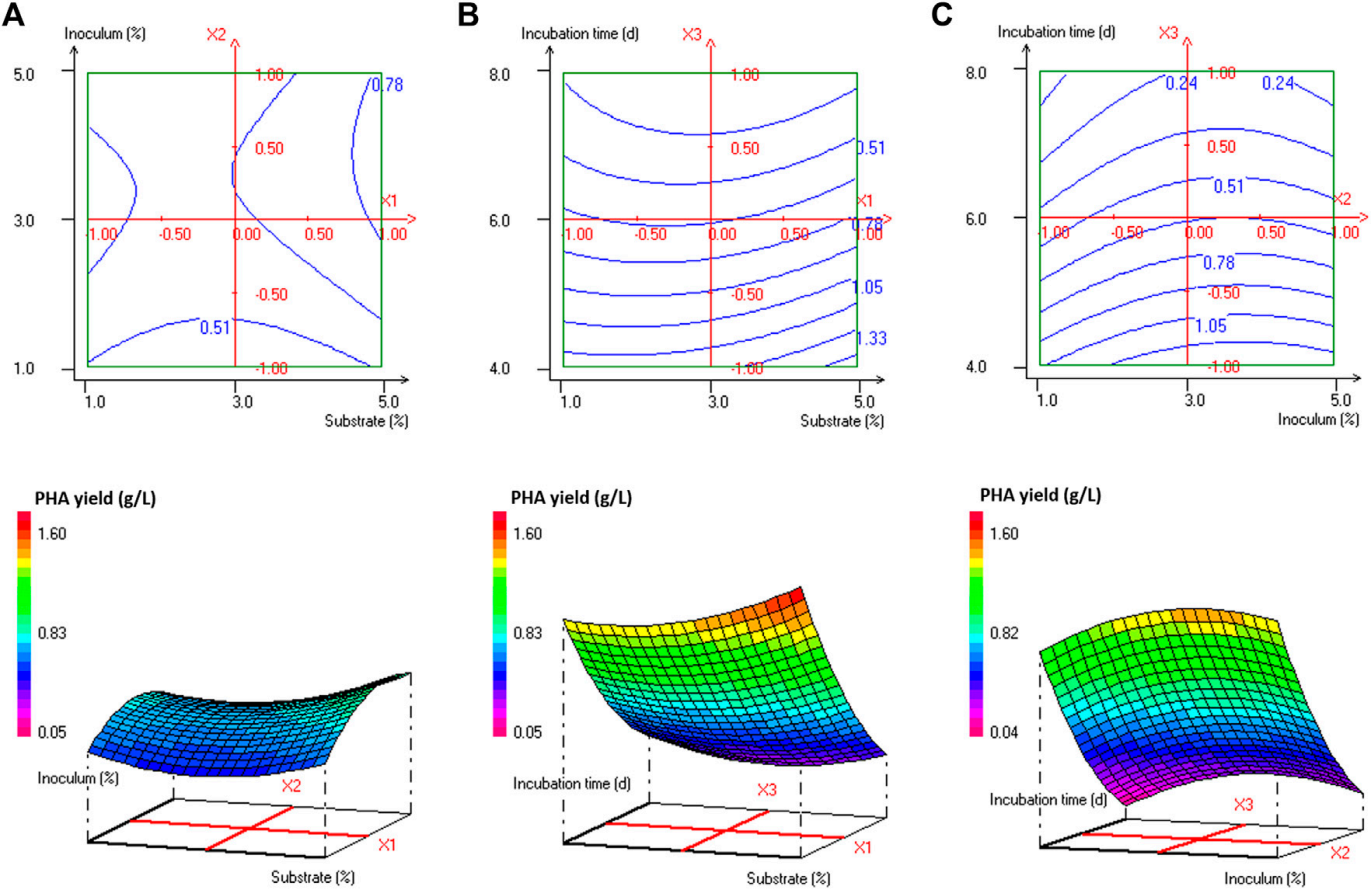
Response surface and contour plots illustrating the effect of **(A)** substrate concentration and inoculum size at 6 days’ incubation time, **(B)** substrate concentration and incubation time at 3% inoculum size, and **(C)** inoculum size and incubation time at 3% substrate concentration on PHA production (g/L) by *H. desertis* G11 using glycerol as substrate.

Analysis of variance (ANOVA) results of this quadratic model was presented in Supplementary Table S1. The regression model for PHA production was highly significant with *p*-values < 0.001%. The quality of fit of the generated equation was further confirmed by the values of the coefficient of multiple determinations (R^2^) and the adjusted coefficient of multiple determinations (Adj. R^2^) of 0.989 and 0.968, respectively. As reported by Nygaard et al. (2019), the experimental design model has a very high fit correlation between observed and predicted response values if R^2^ > 0.9 and a high fit correlation if 0.7 < R^2^ < 0.9. The statistically significant model terms for *H. desertis* PHA production, are the linear regression coefficients (e_2_ and e_3_) and the quadratic regression coefficients (e_11_, e_22_, and e_33_) (Supplementary Table S2). The contour plots and response surface curves for the predicted PHA production yields were shown in Figure 2. They provided useful information about interactions between factors on the PHA accumulation and allowed an easy prediction of the optimal levels of each factor for maximum PHA production.

**FIGURE 3:**
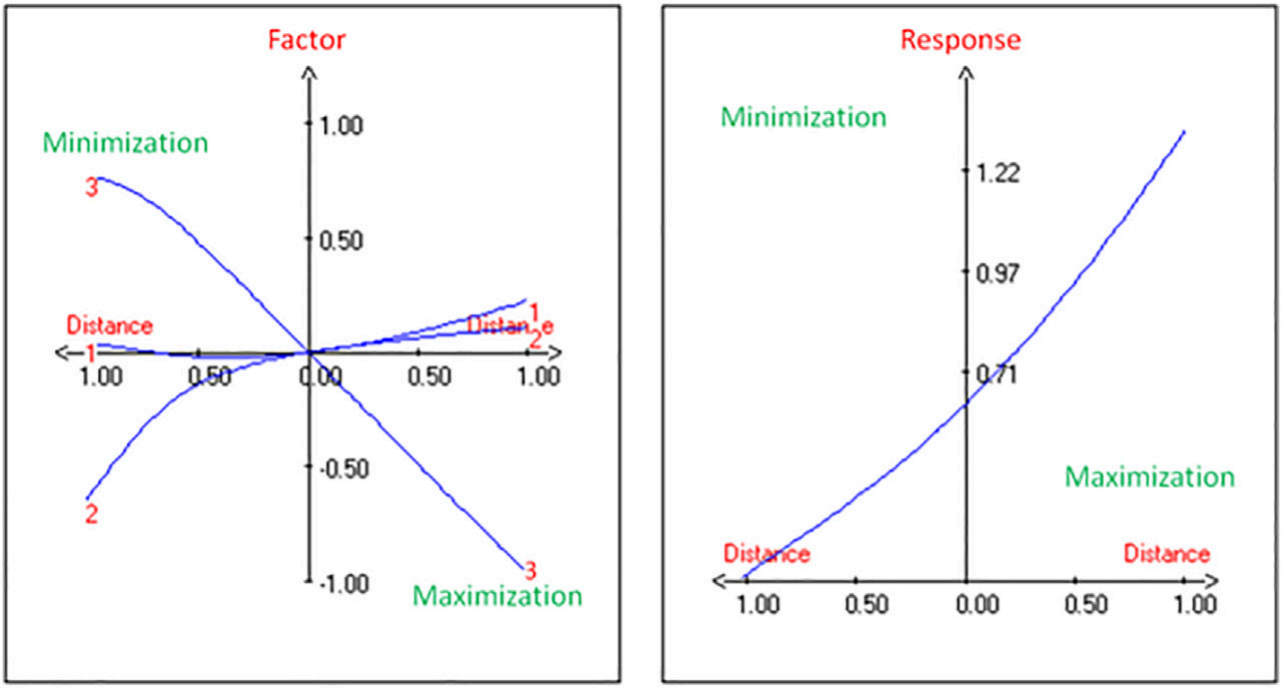
Determination of maximum PHA yield from *H. desertis* G11 through optimum schemes generated by NemrodW software.

The production of PHA by the strain G11 is enhanced by increasing the inoculum size (Figures 2A,C) and decreasing the incubation time (Figures 2B,C). As a result, based on the response surface and contour plots (Figure 2) as well as the optimal scheme (Figure 3), the optimum operating culture conditions, carried out using NemrodW software, were found to be: PHA production of 1.54 ± 0.07 g/L obtained with 5% of glycerol concentration, 4 days for incubation time and 3% of inoculum size. These optimal conditions for PHA production correspond to a biomass production of 2.25 ± 0.37 g/L. Supplementary experiments were carried out under the selected optimal conditions. They led to PHA and biomass productions equal to 1.51 ± 0.06 and 2.23 ± 0.32 g/L respectively, which were in close agreement with the predicted values.

### Characterization of Recovered PHA

**FIGURE 4:**
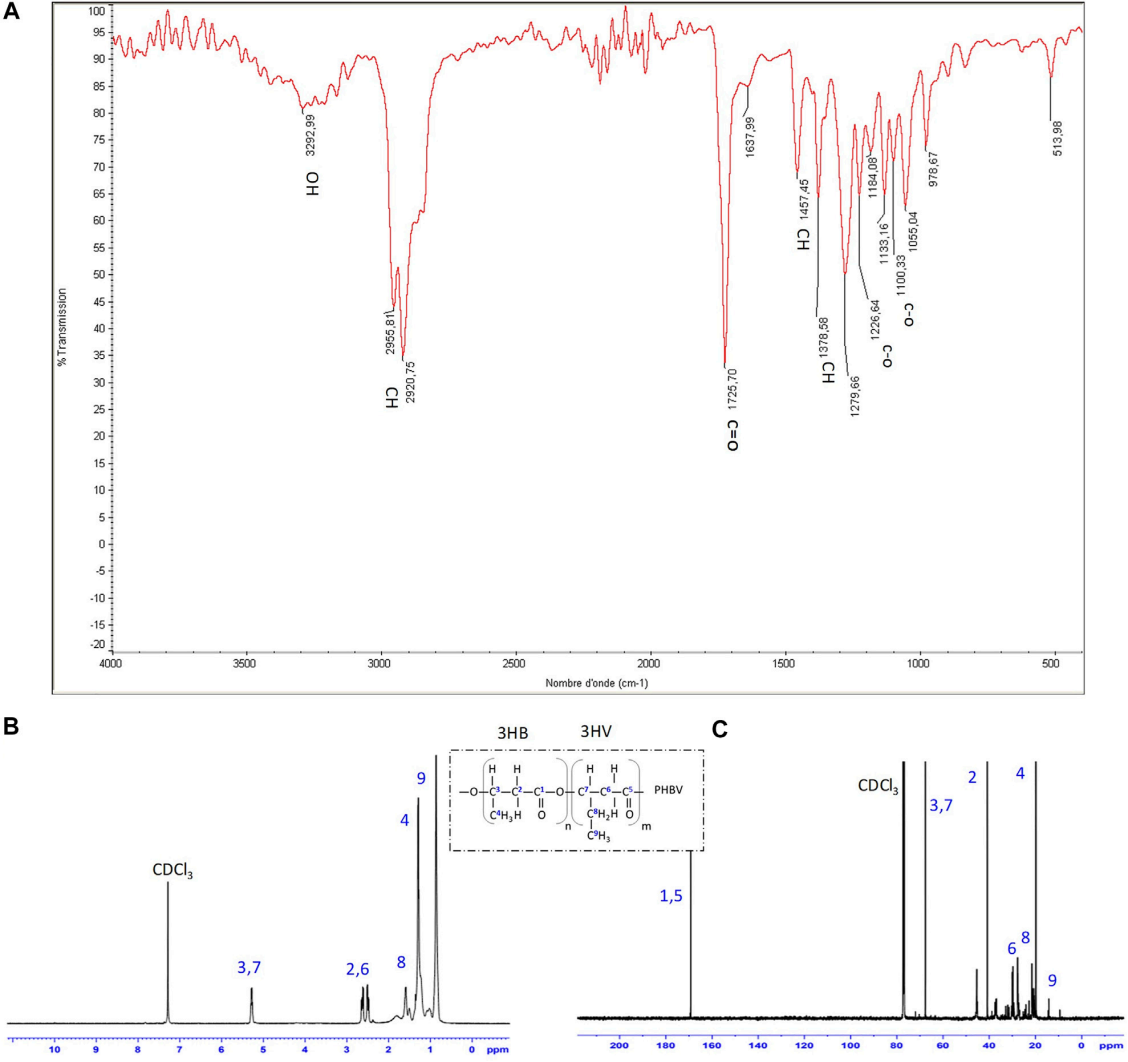
**(A)** FTIR **(B)** 1H-NMR and **(C)** 13C-NMR spectra of purified PHBV isolated from strain G11 grown on glycerol (5%, v/w) as a carbon source.

The presence of the functional groups in the extracted PHA was analyzed by FTIR and NMR spectroscopies (Figure 4) and compared with previous studies (Supplementary Table S3).

FTIR and NMR spectra of PHA produced by *H. desertis* G11 corresponded to a copolymer, PHBV. The FTIR spectrum of PHBV showed an absorption peak at approximately 1,279.66 cm^−1^ corresponding to the saturated ester linkage of C-O-C groups. The absorption peak at 1,378.58 cm^−1^ and 1,457.45 cm^−1^ corresponded to the respective stretching and bending mode of the vibration of the methyl (–CH_3_) group. The peaks at 2.920.75/2955.81, 1725.70, and 3,292.99 cm^−1^ were the respective characteristic peaks of methane (–CH), ester carbonyl (C=O), and hydroxyl (–OH) groups (Figure 4, Supplementary Table S3).

The proton and carbon nuclear magnetic resonance analysis (Figure 4, Supplementary Table S3) confirmed that this indigenously synthesized polymer was composed of 3-hydroxybutyrate (3-HB) and 3-hydroxyvalerate (3-HV) monomers. The chemical shifts of the major signals assigned to the different types of proton atoms [CH_3_ (HB) at 1.253 ppm, CH_3_ (HV) at 0.878 ppm, CH_2_ (HV) at 1.606 ppm, CH_2_ (HV-HB main) at 2.307 ppm, and CH (HV and HB bulk structure) at 5.340 ppm] and carbon atoms [C=O (HB) at 169.12 ppm, C=O (HV) at 169.29 ppm, CH2 (HB) at 40.75 ppm, CH_2_ (HV) at 38.75 ppm, CH (HB) at 67.4 ppm, CH_3_ (HB) at 19.74 ppm and CH_3_ (HV) at 9.31 ppm] in the PHBV structure agree with those obtained by Kemavongse et al. (2008) and showed not only uniformity in results but also a substantial degree of purity in terms of peaks for the extracted PHBV. The molar percentages of HB and HV units in *H. desertis* PHA copolymer were 47.96% and 52.04%, respectively.

### Identification and Evolutionary Analysis of PHA Relevant Genes From *H. desertis* G11

**FIGURE 5:**
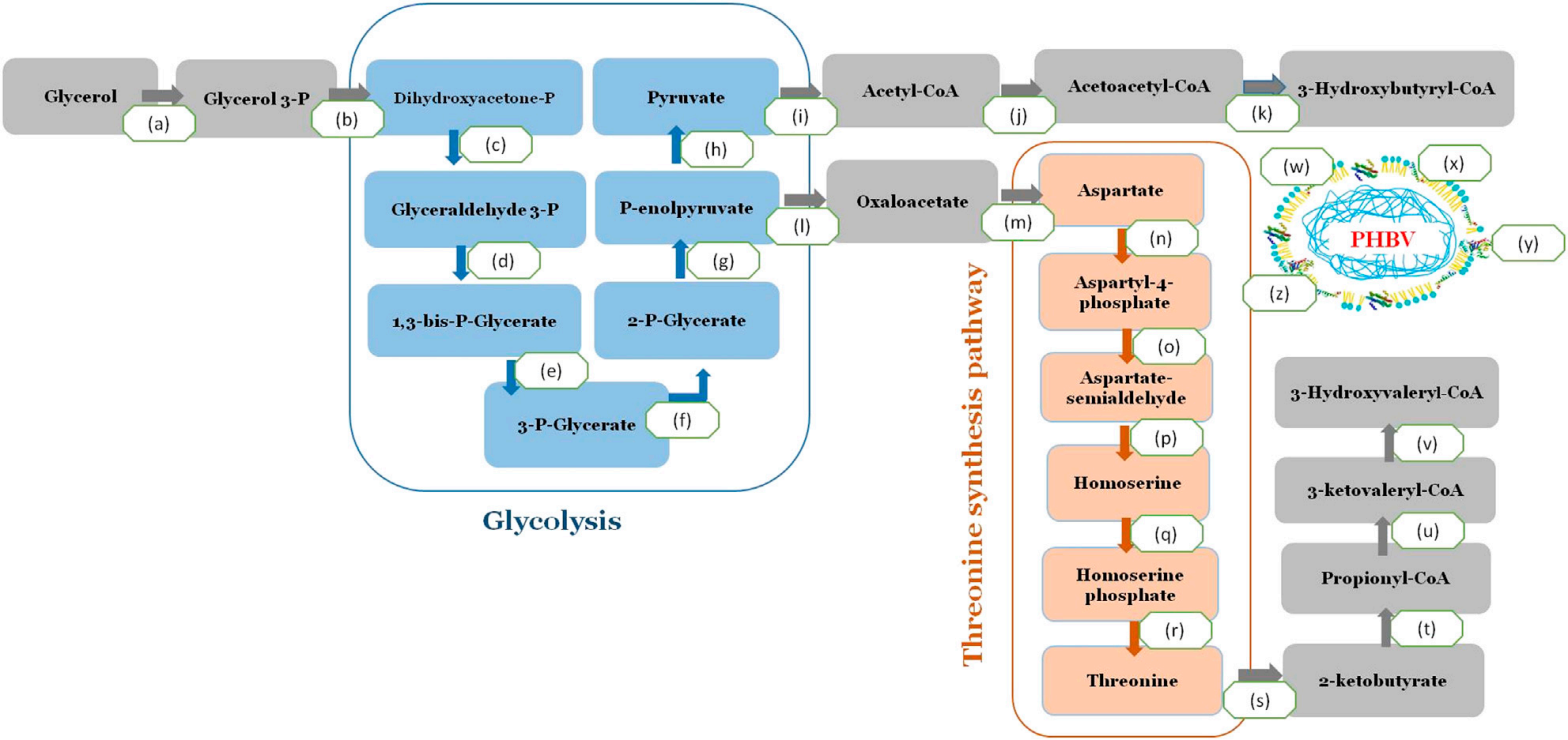
A complete pathway for converting glycerol to PHBV in *H. desertis* genome. (a) Glycerol kinase, EC 2.7.1.30, glpK, A0A1A0F938, (b) Glycerol-3-phosphate dehydrogenase, EC 1.1.5.3 (A0A1A0F0A7) (c) Triosephosphate isomerase, EC 5.3.1.1 (A0A1A0FLG5) (d) Glyceraldehyde-3-phosphate dehydrogenase, EC 1.2.1.12, (A0A1A0FJ82, A0A1A0FBT9, A0A1A0FMC5, A0A1A0F5L9) (e) Phosphoglycerate kinase, EC 2.7.2.3 (A0A1A0F555) (f) 2,3-bisphosphoglycerate-independent phosphoglycerate mutase, EC 5.4.2.12 (A0A1A0FLZ7) (g) Enolase, EC 4.2.1.11 (A0A1A0EWM0), (h) Pyruvate kinase, EC 2.7.1.40 (A0A1A0EQ50, A0A1A0FJ83) (i) Pyruvate dehydrogenase, EC 1.2.4.1 (A0A1A0FHI0) (j) 3-ketoacyl-CoA thiolase (phaA), EC 2.3.1.16 (A0A1A0FJC6) Acetyl-CoA acetyltransferase (EC 2.3.1.9) (A0A1A0FLF6, WP_066316105.1) (k) acetoacetyl-CoA reductase EC 1.1.1.36 (phaB) (A0A1A0FCW9, WP_066319309.1), (l) Class I poly(R)-hydroxyalkanoic acid synthase, EC 2.3.1-(phaC) (A0A1A0ERQ1), (m) Polyhydroxyalkanoate depolymerase (EC 3.1.1.75/76) (A0A1A0FL46) (PhaZ), (n) Phasin family proteins (A0A1A0ERX1, A0A1A0FMH7, A0A1A0EQ92A0EQ92) and (o) Polyhydroxyalkanoate synthesis repressor (PhaR) (A0A1A0EWK0), (p) Homoserine dehydrogenase EC 1.1.1.3, (q) homoserine kinase EC 2.7.1.39, (r) threonine synthase EC 4.2.3.1, (s) threonine deaminase EC 4.3.1.19.

The reported whole-genome sequence of *H. desertis* G11 was used for gene annotations using the RAST server’s rapid annotation platform. The relevant genes for PHA production were identified through homologous alignments against the public annotation databases using BLAST program. A complete pathway for converting glycerol to PHBV was identified in the G11 genome (Figure 5). After a cascade of metabolizing enzymes involving the conversion of glycerol to acetyl CoA, the formation of PHBV from its precursors, acetyl-CoA and propionyl-CoA was involved three enzymes (PhaA, PhaB and PhaC1). *In H. desertis* G11 cells, glycerol can be consumed and metabolized to propionyl-CoA *via* the threonine biosynthetic pathway and subsequently converted to the 3HV monomer (Figure 5). Ketothiolase (PhaA) catalyzed the condensation of two acetyl-CoA or condensation of acetyl-CoA and propionyl-CoA. The resulting intermediates were reduced to 3-hydroxybutyryl-CoA and 3-ketovaleryl-CoA by NADPH dependent acetoacetyl-CoA reductase (PhaB). The hydroxyl acyl-CoA monomers were then incorporated into the growing biopolymer chain by PHA synthase (PhaC1).

Besides genes that are directly involved in PHA biosynthesis, other genes of PHA metabolism were annotated in the genome of G11 strain such as the PHA depolymerase (phaZ) that degrades PHA granules for subsequent reuse, the non catalytic PHA granule associated proteins, phasins that regulate the number and size of the PHA granules and the transcriptional repressor protein, PhaR, that regulates expression of phasin genes and releases repression upon binding PHA.

### *In Silico* Study of PHA Synthase PhaC1 of *H. desertis* G11

**FIGURE 6:**
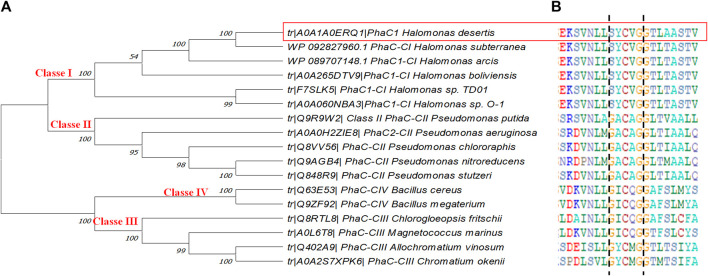
**(A)** Phylogenetic tree of PhaC1 from *H. desertis* G11 with reported PHA synthases. The tree was constructed using Mega-X software. **(B)** Multiple alignments of partial amino acid sequences of PHA synthases exposing lipase box-like patterns from different bacterial species. All the sequences are available on the Universal Protein Resource (UniProt) database. Highlighted sequences correspond to PhaC1 of *H. desertis* G11.

PHA synthase is the crucial enzyme for PHA biosynthesis which polymerizes monomeric hydroxyalkanoate substrates into PHA. They are classified into four classes based on their substrate specificity and subunit composition (Zou et al., 2017). In this study, a phylogenetic tree was constructed based on the amino acid sequences of PhaC1 from *H. desertis* G11 with other bacterial PHA synthases described in the literature and included in the Uniprot database. Analysis of PhaC1 of the strain G11 showed that this enzyme was consistent with class I PHA synthases from other *Halomonas* species (Figure 6A).

Predicted amino acid composition (615 amino acid residues) of PhaC1 from *H. desertis* G11 showed 135 charged amino acids, 81 acidic (Asp and Glu), and 54 basic amino acids (His, Arg, and Lys), respectively. The molecular weight of the protein was estimated at 69.67 kDa with a pI of 4.93. SOPMA analysis indicates that the predicted PHA synthase I protein possesses 45.04% alpha-helices, 12.85% extended strand, 6.50% Beta turn and 35.61% Random coil. Scratch Protein Predictor analysis suggests that the protein is globular and predicted one disulfide bridge formed (2 Cys residues at position 324 and 335) contribute to the structural stability of the protein.

The alignment of amino acid sequences of selected PHA synthases have been allowed the identification of lipase box-like patterns composed of Serine-Tyrosine-Cysteine-valine-Glycine (S-Y-C-V-G) (Figure 6B). *Halomonas* strains have a similar sequence (S-X-C-X-G) at the active site where X is an arbitrary amino acid.

**FIGURE 7:**
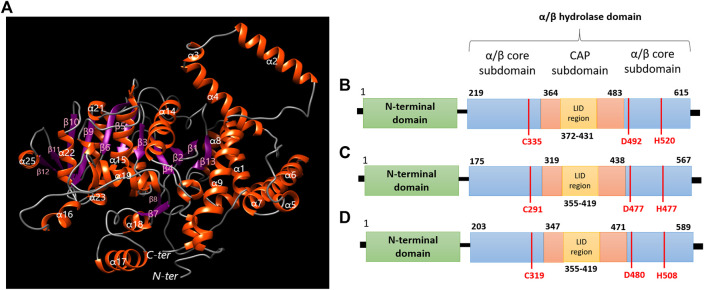
**(A)** Predicted 3D structure modeling information of polyhydroxyalkanoate synthases of *H. desertis* G11 generated by Phyre2 server. **(B)** Domain organization of class I PhaCHd from *H. desertis* G11 compared to **(C)** PhaCCs from *Chromobacterium* sp. USM2 and **(D)** PhaCCn from *Cupriavidus necator*.

Through a structural analysis, PhaC1 of *H. desertis* G11 is folded into a globular structure which belongs to the α/β hydrolase super-family and comprises the N-terminal domain which is important for stabilizing dimeric PhaC and the C-terminal catalytic (CAT) domain-containing the CAP subdomain (residues 364–483) and α/β core subdomains which possesses a catalytic pocket comprising of a catalytic triad (Cys-Asp-His) at its core (Figure 7A). The enzyme composed of 13 β-strands (β1 (Gly230-Asn236), β2 (Leu238-Tyr244), β3 (Pro255-Val250), β4 (Val288-Trp290), β5 (Val329-Tyr334), β6 (Val357-Met363), β7 (Arg400-Ala403), β8 (441-Ala446), β9(Ser484-Lys490), β10(Ala511-Gly517), β11 (Gly534-Thr537), β12 (Thr553-Glu550), β13(Val593-Ala597)) and 28 α-helices (α1 (Glu16-Leu39), α2 (Ser50-Met67), α3 (Leu72-Gln75), α4 (Thr76-Ala96), α5 (Lys114-Thr119), α6 (His123-Asp143), α7 (Ser146-Met167), α8 (Asn176-Glu185), α9 (Asn190-Ala206), α10 (Tyr 266-Leu271), α11 (Gln273-Ser276), α12 (Met277-Gln284), α13 (Pro298-His301), α14 (Trp305-Cys324), α15 (Cys335-Arg352), α16 (Asp374-Asn380), α17 (Leu414-Glu428), α18 (Phe432-Thr439), α19 (Gly447-Glu459), α20 (Lys461-Glu461), α21 (Leu477-Ile480), α22 (Lys496-Leu505), α23 (Gly519-Val525), α24 (Pro528-Lys530), α25 (His545-Thr551), α26 (Trp560-Asn570), α27 (Arg600-Lys603), α28 (Pro607-Glu612)). The core subdomain comprises 13 strands (β1-β13) and 8 helices (α1-α8). The CAP subdomain is connected from β7 and back to the core domain through β8. The catalytic triad (Cys335, His520, and Asp492) is covered by the CAP subdomain which blocks the substrate access through the lid loop structure (a helix-loop-helix motif) (Figure 7B). Once the acyl-CoA enters the catalytic pocket of the enzyme, the catalytic His activates the nucleophilic Cys, which subsequently attacks the thiol group of acyl-CoA. The catalytic Asp is proposed to activate the 3-OH group of acyl-CoA to attack the second incoming substrate for the elongation process (Yuan et al., 2001; Tian et al., 2005; Wahab et al., 2006).

## Discussion

Halophilic microorganisms especially *Halomonas* spp., have been attracted a lot of interest, recently due to their potential to produce exoenzymes, exopolysaccharides, osmolytes, biosurfactants, and bioplastics (Neifar et al., 2019). In this work, we report the capacity of the hydrocarbonoclastic, plant growth-promoting halophilic bacterium *H. desertis* G11 (Naili et al., 2018; Neifar et al., 2019; Riahi et al., 2020) to produce PHA. The ability of this strain to grow at NaCl concentrations up to 20% with an optimum at 5%–10% allowing an open and continuous fermentation process without contamination and to produce many compatible organic solutes such as osmoregulated periplasmic glucans, choline-glycine betaine, and ectoine hydroxyectoine make it a well-equipped bacterium to cope with the osmotic stress (Neifar et al., 2019).

In this study, the potential of halophile *H. desertis* G11 to produce PHA was firstly revealed using Nile bleu A staining plate assay (Ostle and Holt, 1982) under saline condition (5% NaCl). The bright pink fluorescence displayed by the bacterial colonies under UV light indicates intracellular accumulation of PHA by G11. Although the mechanism of selective PHA staining by Nile blue is not yet fully understood, it has been suggested that the dye diffuses through the bacterial membrane and labels cytoplasmic PHA granules by binding to lipid molecules in the surrounding layer. Thus, this method has been adapted for many years, for its affinity to bind to PHA granules and has therefore been accepted as a presumptive test for PHA storage in bacteria. This method has been applied to other strains such as *Bacillus subtilis* MANA18 (Al-Hamdani and Jaber, 2017) and *Vibrio* sp. BTTC26 (Raghul. 2012). The *Halomonas* PHA granules have a diameter range of 0.2–0.5 µM as reported by Muhammadi et al. (2015) and Obruca et al. (2020). The use of lipophilic dye staining and TEM and SEM microscopy techniques have been applied to detect PHA granules in other *Halomonas* species including *Halomonas* sp. SF2003, *H. boliviensis* LC1, *Halomonas* sp. TD01, *H. pacifica* ASL10 and *H. salifodiane* ASL11 and *H. hydrothermalis* (Quillaguaman et al., 2006; Fu et al., 2014; Thomas et al., 2019; El-malek et al., 2020; Obruca et al., 2020).

PHA production by *H. desertis* G11 was assayed in submerged cultures using glycerol as a substrate. Statistical analysis and model fitting of the optimization of PHA production was performed using BBD and RSM. The optimum PHA production (1.5 g/L; 68% of DCW) was obtained at 4 days while longer incubation time showed decreasing trend. Incubation period for PHA production by different organisms reported so far was longer than 24 h (Kulkarni et al., 2010). *H. boliviensis* required 33 h incubation period for highest PHA accumulation (Quillaguaman et al., 2006). Other species of *Halomonas* such as *H. profoundus*, *H. elongate* and *Halomonas marina* required 3 days for PHA production (Mothes et al., 2008; Simon-Colin et al., 2008; Biswas et al., 2009). In contrast, a longer incubation time of 120 h was required by the halophilic strain *H. elongata* to produce similar PHA content (50–60 wt%) (Mothes et al., 2008; Biswas et al., 2009).

Halophiles are of particular interest since they eliminate the need to maintain aseptic conditions and enable continuous production in saline (even sea) water and they serve as feasible genetic sources for industrially important compounds like biopolymers, osmolytes and extremozymes (Chen et al., 2018; Rodriguez-Perez et al., 2018). Several halophilic microorganisms demonstrated their ability to produce PHA. Most of them belong to the family *Halomonadaceae* (Haba et al., 2011). The genus *Halomonas* is known to accumulate scl-PHA (Table 2). Most *Halomonas* sp. has a NaCl requirement of 3%–15% for optimal growth and this concentration is sufficient enough to prevent microbial contamination. *Halomonas* species including *H. marina*, *H. boliviensis*, *H. hydrothermalis*, *Halomonas* sp. *TD01*, *H. elongata* DSM 2581, and *H. campaniensis*, isolated from different saline habitats in the word, have been reported as high polymer accumulators from different sugars, sodium acetate and butyric acid, and agricultural residues (Mothes et al., 2007; Van-Thuoc et al., 2008; Biswas et al., 2009; Quillaguamán et al., 2010; Tao et al., 2017; Cristea et al., 2018).

**TABLE 2:** Production of PHA by halophilic or halotolerant *Halomonas* strains.

Strategy	Strain	Carbon source	Type of PHA	PHA composition (mol%)		PHA content (g/L)	PHA (w%)	References
				3HB	3HV			
Wild strains	*H. elongata* 2FF	Glucose	PHB	—		0.95	40	Cristea et al. (2018)
	*H. elongata* DSM 2581	Glucose	PHB	—		2.2	32	Ilham et al. (2014)
	*H. halophila* CCM 3662	Glucose	PHB	—		1.48	45.5	Kucera et al. (2018)
	*H. campisalis* MCM B-1027	Maltose	PHBV	96.4	3.6	ND	44	Kulkarni et al. (2011)
	*H. bluephagenesis* TD01	Glucose	P (3HB-co-4HB)	—		50	61	Chen and Jiang, (2017)
	*Halomonas* TD01	Glucose	PHB	—		4.14	69	Tan et al. (2011)
	*H. vesnusta* KT832796	Glycerol	PHB	—		0.374	33.12	Stanley et al. (2018)
	*H.* sp.KM-1	Glycerol	PHB	—		ND	48	Kawata and Aiba, (2010)
	*H. profundus*	Glycerol	PHB	—		ND	ND	Simon-Colin et al. (2008)
	*H. desertis* G11	Glycerol	PHBV	—		1.5	68.18	This work
Engineered strains	*H. bluephagenesis*	Glucose	PHBV	—	2.38	ND	ND	Chen et al. (2019)
	*H.* TD01	Glucose/Glycerol/maltose	PHBV	—	4	ND	ND	Tan et al. (2014)
Precursors addition	*H. profundus*	Glycerol + valerate	PHBV	61	39	ND	ND	Simon-Colin et al. (2008)
	*H. profundus*	Glycerol + propionate	PHBV	88	12	ND	ND	Simon-Colin et al. (2008)
	*Halomonas* sp. O-1	Glucose + sodium valerate	PHBV	72	28	ND	ND	Ilham et al. (2014)

The use of glycerol as a substrate allows higher PHBV content with reduced cost, it does not consume phosphoenolpyruvate of the PTS transporter system compared with other sugars, more oxaloacetate and energy can be saved for PHBV production (Jincy et al., 2013; Tan et al., 2014). As crude glycerol contains high salt concentration, ranging from 3 to 7% w/v, the possibility of using halophilic bacteria could potentially provide an attractive production system for PHA (Van-Thuoc et al., 2015). Glycerol has previously been reported as an ideal carbon source for PHA production in some *vibrios* (Chien et al., 2007; Wei et al., 2011; Raghul. 2012), *Ralstonia eutropha* (Taidi et al., 1994). In addition, results with *Bacillus megaterium* demonstrated the valorization of glycerol for PHB production (Naranjo et al., 2013). They were able to produce 4.8 g/L of PHB using 2% purified glycerol under controlled conditions. Jincy et al. (2013) performed statistical optimization for PHB production (0.60 g/L) using 2% crude glycerol by *Bacillus firmus* NII 0830. Studies also revealed the ability of several *Halomonas* strains to accumulate PHA from glycerol among them, *H. campisalis* ATCC 700597T synthesizes PHB with a yield of 24.4%, *H. meridiana* NBRC 15608T which gives about 25% by weight of PHB. Similarly, *Halomonas sp*. KM-1 accumulates about 34% of PHB and 63.6% at 3% and 10% glycerol respectively (Kawata and Aiba, 2010). More recently, Dubey and Mishra (2021) reported a PHA content of 0.20 ± 0.02 g/100 ml in *H. daqingensis* and 0.21 ± 0.01 g/100 ml in *H. ventosae* when cultivated in 3% algal biodiesel waste residue, 5% NaCl supplementation at 35°C within 48 h of incubation. These result points to the future use of a cheap industrial by-product such as glycerol from biodiesel waste in the industrial production of PHA by the halophilic *Halomonas* species.

The chemical structures of the purified polymer were analyzed by FT-IR and ^1^H and ^13^C NMR. The assigned signals in the corresponding spectra agree with earlier studies (Kemavongse et al., 2008; Shamala et al., 2009; Kulkarni et al., 2011; Getachew and Woldesenbet, 2016; El-malek et al., 2020), confirming the chemical structure of PHBV. Based on the peak areas of the CH_3_ of HB unit and the CH_3_ of HV unit, the mole fraction of 3HV was determined as 52.04 mol %. It has been reported that *Halomonas* species can accumulate PHB, PHBV and poly (3-hydroxybutyrate-co-4-hydroxybutyrate) (P3HB4HB) (Ren et al., 2018; Chen et al., 2019; Ye et al., 2020). The copolymer PHBV is of high commercial interest due to its favorable properties compared to PHB (Tian et al., 2001; Chen. 2009; Ferre-Guell and Winterburn, 2018). Most PHBV producers require co-substrates such as propionic acid or valeric acid. *H. profundus* produces PHBV with different molar fractions of 3HV in the presence of valeric acid and propionic acid (Simon-Colin et al., 2008). Similarly, a prerequisite to the formation of 3HV-CoA in *E. coli* is the intracellular presence of propionyl-CoA as a precursor (Srirangan et al., 2016). The supplementation of precursors due to the lack of intracellular propionyl-CoA as a precursor in the biotransformation systems is toxic which not only inhibit cell growth but also increase production cost but also makes PHBV production difficult to control and thus increases production cost (Steinbüchel and Lutke-Eversloh, 2003). CRISPR/Cas9 method was used to engineer the TCA cycle in *H. bluephagenesis* on its chromosome for production of PHBV from glucose as a sole carbon source (Yu et al., 2020). The recombinant *Halomonas* TD08 is able to produce PHBV consisting of 4–6 mol% 3HV, from various carbohydrates as the sole carbon source (Tan et al., 2014). The copolymer formation without addition of precursors or genetic engineering tools has been reported in a few microorganisms such as *Bacillus cereus* SPV, *Rhodobacter rubber*, *Nocardia corallina*, *Cupriavidus necator* SH-69, *Agrobacterium* sp. strain SH1 and GW-014, *Rhodospirillum Rubum*, and *H. campisalis* MCM B-1027 (Valappil et al., 2008; Kulkarni et al., 2010; Policastro et al., 2021). To our knowledge, the moderately haloalkalitolerant strain, *H. campisalis* isolated from alkaline and saline Lonar Lake (Buldhana India) was the only *Halomonas* strain reported to accumulate PHBV (10.4 mol% HV) using a simple carbon source without addition of any precursors (Kulkarni et al., 2010).

The metabolic pathways relevant to glycerol assimilation and PHBV biosynthesis was predicted by genome analysis of *H. desertis* G11. In the predicted pathways, glycerol can be metabolized to two essential precursors acetyl-CoA through glycolysis pathway and converted to the 3HB monomer and to propionyl-CoA via threonine biosynthetic pathway and converted to 3HV monomer. These precursors lead to PHBV formation by three crucial enzymes. The genes encoding these three key enzymes of PHBV biosynthesis (PhaA, PhaB, and PhaC) are frequently organized in a single operon, as in *C. necator* or *Pseudomonas* sp. (Babel et al., 2001; Luengo et al., 2003). This organization is absent in other bacteria such as *Halomonas* sp. SF 2003. Idem, the genes encoding the three key enzymes of PHA biosynthesis pathway are not clustered in an operon in G11 strain genome but are scattered all over the genome. It has been reported that the organization of the biosynthetic genes depends on the bacterial species and also on the class of PHA synthases.

Based on phylogenetic analyses performed in this study, PhaC1 present in the genome of *H. desertis* G11 belongs to class I. Similar results were found with *Halomonas* sp. TD01 (Cai et al., 2011), *Halomonas* sp*.* SF2003 (Thomas et al., 2020) and *H.* sp. O-1 (Ilham et al., 2014). Multiple alignments of PHA synthases of several *Halomonas* species allowed identifying different PhaC box consensus sequences (Thomas et al., 2020). Analysis of CI-PhaC amino acids sequences of different halophiles PHA-producing *Halomonas* strains showed similar lipase box-like sequence (Ser-X-Cys-X-Gly) (figX). From the database analysis the most common described pattern is Glycine-X-Cysteine-X-Glycine (G-X-C-X-G) which can be modified to (GS)-X-C-X-(GA)-(GA) (Nambu et al., 2019). The PhaC_Hd_ has a lipase box-like sequence, S-X-C-X-G located between the residue 371 and the residue 375.

Structural analysis by different web servers for protein modeling (Phyre2, iterative TASSER, Swiss-Model), generated only two reliable three-dimensional protein structure models for Pha C1 of *H. desertis* G11 (Figure 7). The abilities of both strains *Chromobacterium* sp. USM2 and *C. necator* to produce PHBV from different carbon sources, have been previously reported (Obruca et al., 2010; Aramvash et al., 2016; Chek et al., 2017). Obruca et al. (2010) investigated the production of PHBV by *Cupriavidus necator* H16 in fed-batch mode from waste rapeseed oil using propanol as a precursor of 3-hydroxyvalerate. Aramvash et al. (2016) studied the effects of using a combination of substrates (fructose, propanol, citric acid, acetic acid, propionic acid and beef extract) on the PHBV production. The results of batch PHBV optimization by RSM, indicated that fructose in combination with propanol and beef extract, showed better PHBV yield, as compared to that of propionic acid. Chek et al. (2017) published a high-resolution crystal structure of the catalytic domain of homodimer Class I PHA synthase from *Chromobacterium* sp. USM2. This enzyme produces poly (3-hydroxybutyrate-co-3-hydroxyhexanoate) copolymer and poly (3-hydroxybutyrate-co-3-hydroxyvalerate-co-3-hydroxyhexanoate) terpolymer, which are good materials for industry due to their softness and flexibility (Jia et al., 2000; Amara and Rehm 2003; Tian et al., 2005).

## Conclusion

The production of PHA by the halophilic strain *H. desertis* G11 on glycerol-based medium under saline condition was successfully optimized through statistical approach (BBD and RSM) indicating that G11 is an efficient PHA producer (1.5 g/L; 68% of DCW) with a high potential for industry applications. The NMR and FTIR spectra showed the presence of required functional groups in biosynthesized PHBV based on chemical shifts and bands observed. Genomic analysis revealed the presence of the key genes involved in the biosynthesis and biodegradation of PHA including *phaA*, *phaB*, *phaC*, *phaZ*, *phaP* and *phaR*. Through multiple sequences alignments with its homologs, *H. desertis* PhaC1 can be classified as a class I PHA synthase. Threonine pathway seems to have a key role in PHV synthesis.

The promising potential to use crude glycerol as low-cost substrate, in addition to its capacity to grow in front of stressful conditions and to produce PHA copolymers, make *H. desertis* G11 a versatile strain with a high potential for biotechnological use. The future research will be focused on 1) scaling up of the optimized process in bioreactor (fed batch and continuous processes) using biodiesel waste as carbon substrate; 2) studying the thermomechanical and visco-elastic properties of produced PHA; 3) valorizing low-cost substrates for the production of PHA under non-sterile high-salting conditions, 4) studying the role of PHA synthase in the synthesis of PHBV by *H. desertis* G11 using X-ray crystallography and exploiting the *phaC* gene for genetic and enzyme engineering, and 5) developing suitable genetic tools for manipulating the *H. desertis* G11 as a platform for synthesizing polymers.

## Data Availability

The datasets presented in this study can be found in online repositories. The names of the repository/repositories and accession number(s) can be found in the article/Supplementary Material.
