# Supplementary material for: Extremophilic Bacterium Halomonas desertis G11 as a Cell Factory for Poly-3-Hydroxybutyrate-co-3-Hydroxyvalerate Copolymer’s Production
Source: Front Bioeng Biotechnol. 2022 May 23;10:878843. doi: 10.3389/fbioe.2022.878843 (PMC9168272; doi:10.3389/fbioe.2022.878843)
Supplement: Supplementary file 1 [file Table1.DOCX]

**Table S1.** Analysis of variance (ANOVA) for the response surface quadratic model for PHA production by *H. desertis* G11.

| **Source of variation** | **Sum of squares** | **Degrees of freedom** | **Mean square** | ***F*-Ratio** | **Significance*** |
| --- | --- | --- | --- | --- | --- |
| Response 1: PHA production (g/L) R^2^ = 0.989 R^2^A=0.968 | | | | | |
| Regression | 2.5282 | 9 | 0.2809 | 48.1556 | *P* ˂0.05 |
| Residuals | 0.0292 | 5 | 0.0058 |  |  |
| Lack of fit | 0.0225 | 3 | 0.0075 | 2.2500 | NS |
| Pure error | 0.0067 | 2 | 0.0033 |  |  |
| Total | 2.5573 | 14 |  |  |  |
| Response 2: Biomass production (g/L) R^2^ = 0.969 R^2^A=0.913 | | | | | |
| Regression | 28.9833 | 9 | 3.2204 | 17.3138 | *P* ˂0.05 |
| Residuals | 0.9300 | 5 | 0.1860 |  |  |
| Lack of fit | 0.7500 | 3 | 0.2500 | 2.7778 | NS |
| Pure error | 0.1800 | 2 | 0.0900 |  |  |
| Total | 29.9133 | 14 |  |  |  |

* Levels of significance (*P*) of the ANOVA (NS= non significant)
