# Supplementary material for: Extremophilic Bacterium Halomonas desertis G11 as a Cell Factory for Poly-3-Hydroxybutyrate-co-3-Hydroxyvalerate Copolymer’s Production
Source: Front Bioeng Biotechnol. 2022 May 23;10:878843. doi: 10.3389/fbioe.2022.878843 (PMC9168272; doi:10.3389/fbioe.2022.878843)
Supplement: Supplementary file 2 [file Table2.DOCX]

**Table S2.** Estimates of and statistics on the coefficients.

| **Name** | **Coefficient** | **F. inflation** | **Standard deviation** | **t exp.** | **Significance*** |
| --- | --- | --- | --- | --- | --- |
| Response 1: PHAproduction (g/L) | | | | | |
| e_0_ | 0.633 |  | 0.044 | 14.36 | *P* ˂0.05 |
| e_1_A | 0.062 | 1.00 | 0.027 | 2.31 | NS |
| e_2_B | 0.087 | 1.00 | 0.027 | 3.24 | *P* ˂0.05 |
| e_3_C | -0.525 | 1.00 | 0.027 | -19.44 | *P* ˂0.05 |
| e_11_A^2^ | 0.108 | 1.01 | 0.040 | 2.73 | *P* ˂0.05 |
| e_22_B^2^ | -0.142 | 1.01 | 0.040 | -3.56 | *P* ˂0.05 |
| e_33_C^2^ | 0.133 | 1.01 | 0.040 | 3.35 | *P* ˂0.05 |
| e_12_AB | 0.050 | 1.00 | 0.038 | 1.31 | NS |
| e_13_AC | -0.075 | 1.00 | 0.038 | -1.96 | NS |
| e_23_BC | -0.025 | 1.00 | 0.038 | -0.65 | NS |
| Response 2: Biomass production (g/L) | | | | | |
| e_0_ | 5.000 |  | 0.249 | 20.08 | *P* ˂0.05 |
| e_1_A | 0.000 | 1.00 | 0.152 | 0.00 | NS |
| e_2_B | 0.625 | 1.00 | 0.152 | 4.10 | *P* ˂0.05 |
| e_3_C | -0.500 | 1.00 | 0.152 | -3.28 | *P* ˂0.05 |
| e_11_A^2^ | -0.937 | 1.01 | 0.224 | -4.18 | *P* ˂0.05 |
| e_22_B^2^ | -0.687 | 1.01 | 0.224 | -3.06 | *P* ˂0.05 |
| e_33_C^2^ | -2.187 | 1.01 | 0.224 | -9.75 | *P* ˂0.05 |
| e_12_AB | 0.625 | 1.00 | 0.216 | 2.90 | *P* ˂0.05 |
| e_13_AC | 0.125 | 1.00 | 0.216 | 0.58 | NS |
| e_23_BC | 0.625 | 1.00 | 0.216 | 2.90 | *P* ˂0.05 |

* Levels of significance (*P*) of the ANOVA (NS= non significant).
