# Supplementary material for: Extremophilic Bacterium Halomonas desertis G11 as a Cell Factory for Poly-3-Hydroxybutyrate-co-3-Hydroxyvalerate Copolymer’s Production
Source: Front Bioeng Biotechnol. 2022 May 23;10:878843. doi: 10.3389/fbioe.2022.878843 (PMC9168272; doi:10.3389/fbioe.2022.878843)
Supplement: Supplementary file 3 [file Table3.DOCX]

**Table S3.** Detection of PHBV formed by *Halomonas desertis* G11 using FTIR and NMR (^1^H and ^13^C) spectroscopy.

| **Polymer analysis** | **Wavenumbers (cm^-1^)** | **Assignment** | **Wavenumbers (cm-1) of PHBV from literature**  (Kemavongse et al., 2008; El-malek et al., 2020) |
| --- | --- | --- | --- |
| **FTIR** | 1279.66 | C–O-C | 1300 |
|  | 1378.58/1457.45 | –CH3 | 1379 |
|  | 2.920.75 / 2955.81 | –CH | 2924/3531 |
|  | 1725.70 | C=O | 1725 |
| **NMR spectroscopy** | **Chemical shift (ppm)** | **Assignment** | **Chemical shift (ppm) of PHBV from literature** (Kemavongse et al., 2008; El-malek et al., 2020) |
| **^13^C-NMR** | 169.12 | C=O | 169 |
|  | 40.75 | CH_2_ (HB) | 40 |
|  | 67.4 | CH | 68 |
|  | 19.74 | CH_3_ (HB) | 20 |
|  | 169.29 | C=O | 169 |
|  | 38.75 | CH_2_ (HV) | 38 |
|  | 9.31 | CH_3_ (HV) | 9 |
| **^1^H-NMR** | 0.878 | CH_3_ (HV) | 0.83 |
|  | 1.253 | CH_3_ (HB) | 1.25 |
|  | 1.606 | CH_2_ (HV) | 11.68 |
|  | 2.307 | CH_2_ (HV-HB main) | 2.17 |
|  | 5.340 | CH | 5.22 |
