# Supplementary figures and images for: Extremophilic Bacterium Halomonas desertis G11 as a Cell Factory for Poly-3-Hydroxybutyrate-co-3-Hydroxyvalerate Copolymer’s Production
Source: Front Bioeng Biotechnol. 2022 May 23;10:878843. doi: 10.3389/fbioe.2022.878843 (PMC9168272; doi:10.3389/fbioe.2022.878843)

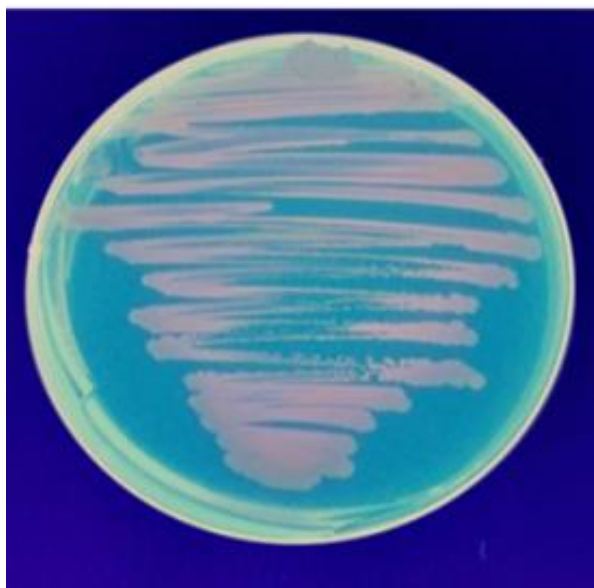

**Figure S1.** Pink florescence under UV light by PHA producer *Halomonas desertis* G11.

Supplement: Supplementary file 4 [file Image1.pdf]
